# The combined fatigue effects of sequential exposure to seated whole body vibration and physical, mental, or concurrent work demands

**DOI:** 10.1371/journal.pone.0188468

**Published:** 2017-12-13

**Authors:** Marcus Yung, Angelica E. Lang, Jamie Stobart, Aaron M. Kociolek, Stephan Milosavljevic, Catherine Trask

**Affiliations:** 1 Canadian Centre for Health & Safety in Agriculture, College of Medicine, University of Saskatchewan, Saskatoon, Saskatchewan, Canada; 2 School of Physical Therapy, College of Medicine, University of Saskatchewan, Saskatoon, Saskatchewan, Canada; 3 School of Physical and Health Education, Nipissing University, North Bay, Ontario, Canada; University of L'Aquila, ITALY

## Abstract

Many occupations in agriculture, construction, transportation, and forestry are non-routine, involving non-cyclical tasks, both discretionary and non-discretionary work breaks, and a mix of work activities. Workers in these industries are exposed to seated whole body vibration (WBV) and tasks consisting of physical, mental, or a combination of demands. Risk assessment tools for non-routinized jobs have emerged but there remains a need to understand the combined effects of different work demands to improve risk assessment methods and ultimately inform ergonomists and workers on optimum work arrangement and scheduling strategies. The objective of this study was to investigate fatigue-related human responses of WBV sequentially combined with physical, mental, or concurrent physical and mental demands. Sixteen healthy participants performed four conditions on four separate days: (1) physically demanding work, (2) mentally demanding work, (3) concurrent work, and (4) control quiet sitting. For each condition, participants performed two 15-minute bouts of the experimental task, separated by 30-minutes of simulated WBV based on realistic all-terrain vehicle (ATV) riding data. A test battery of fatigue measures consisting of biomechanical, physiological, cognitive, and sensorimotor measurements were collected at four interval periods: pre-session, after the first bout of the experimental task and before WBV, after WBV and before the second bout of the experimental task, and post-session. Nine measures demonstrated statistically significant time effects during the control condition; 11, 7, and 12 measures were significant in the physical, mental, and concurrent conditions, respectively. Overall, the effects of seated WBV in combination with different tasks are not additive but possibly synergistic or antagonistic. There appears to be a beneficial effect of seated ATV operation as a means of increasing task variation; but since excessive WBV may independently pose a health risk in the longer-term, these beneficial results may not be sensible as a long-term solution.

## Introduction

Work tasks in manufacturing settings are often predictable; however, jobs in agriculture, transportation, forestry, and construction are typically less routinized, non-cyclical, peripatetic, and involve both discretionary and non-discretionary work breaks [1–3]. In these sectors and in many contemporary jobs, workers are exposed to a mix of work activities, including exposure to seated whole body vibration (WBV) with tasks consisting of physical, mental, or a combination of demands [4]. Unfortunately, conventional exposure and risk assessment methods, which assume regular work cycles to extrapolate into longer time periods, are not well suited to evaluate the risk of musculoskeletal disorders and other adverse health outcomes in non-routinized work [2]. Although risk assessment tools and methods developed for non-routinized jobs have emerged [2], there remains limited information on the combined effects of various work demands and activities on MSD risk. These effects can be documented by measuring the operator’s fatigue, which is operationally defined as a multidimensional construct that involve physical, cognitive, and visual processes, and commences from the start of activity.

Non-routine work such as agriculture has been shown to involve physical demands (e.g., manual material handling, shovelling, pitch fork work, picking and pruning, and overhead work [5–6]). Tasks such as maintenance and handling livestock, also involve mental demands that require routine (i.e., regulated actions that are performed unconsciously and automatically) or active knowledge (i.e., regulated actions performed consciously through established cognitive rules or algorithms) [7]. Agricultural work also consists of frequent exposure to seated WBV from farm equipment and vehicle operation [8]. These primary tasks and demands may occur sequentially; for instance, farmers might operate an all-terrain vehicle quad bike (ATV) to herd/handle livestock or travel to various locations on the farm, and at their destination they might perform repetitive heavy loading and unloading lifting tasks. Strong evidence indicates that physically demanding work such as forceful and repetitive exertions can lead to fatigue effects, which are subsequently related to increased risk of occupational incidents and may be a precursor to musculoskeletal disorders [9]. Mentally demanding tasks have also been linked to elevated risk of occupational accidents [10]. It has been postulated that mental demands may decrease vigilance and increase mental fatigue, leading to increased reaction time, errors, and false alarms [11]. When physical and mental demands are concurrent, the risk of injury is likely exacerbated. For instance, physical capacity is adversely affected by mental demand, resulting in a decrease in endurance time, increased fatigability, quicker rate of strength decrement, and slower heart rate recovery [12]. In another study, significant increases in spine loading were observed with concurrent cognitive and physical tasks; less controlled trunk motion and increase in torso muscle co-activation was attributed to work-related mental processing [13]. Not surprisingly, both physical and mental demands also contribute to decrements in operation system performance, such as increased work error rates [14].

There is strong epidemiological evidence that on its own, prolonged exposure to intense seated WBV can lead to long-term health problems. Whole body vibration may be a risk factor for the development of low back pain, peripheral nervous system dysfunction, visual and vestibular disturbances, prostate disorders, and gastrointestinal problems [15–17]. The acute effects of occupational levels of WBV is less understood and the evidence is inconclusive [18], but has been speculated to be a contributing factor to fatal and non-fatal occupational injury [19], including machinery-related injuries, falls, and vehicle crashes [20–21]. According to Transport Canada, 19.5% of all road fatalities between 2003 and 2007 were a result of heavy vehicle collisions, a vast majority of crashes were attributed to driver error [22]; it has been speculated that these errors may be caused or compounded by the effects of WBV [23]. Thus, there is preliminary evidence that both seated WBV and physical and mental work demands independently pose a risk of both acute incidents and long term health effects. However, the effects of seated WBV exposure combined with mental or physical work tasks remain unclear.

By investigating the combined effects of different work demands, we can better understand whether fatigue effects of sequential demands are additive, synergistic, multiplicative, or antagonistic; such findings would be an initial step towards informing health and safety practitioners, ergonomists, and workers to develop effective risk assessment techniques and ultimately work arrangement or scheduling strategies. The objective of the present study was to investigate fatigue-related human responses of seated WBV, when sequentially combined with physically demanding, mentally demanding, and a concurrent physical and mental demanding task.

## Methods

### Participants

Sixteen healthy participants (Table 1) from the university community were recruited to perform four conditions combined with WBV. Each condition was separated into four sessions, at least 24 hours apart, with the order randomized for each participant. Participants were asked to refrain from exercise and caffeine/alcohol consumption 24 hours prior to all experimental sessions. All participants provided written consent to the terms and conditions of the study, including the procedures, possible risks, and photo release authorization. This study was approved by the Research Ethics Board of the University of Saskatchewan. The individual in this manuscript has given written informed consent (as outline in PLOS consent form) to publish these case details.

**Table 1:** Participant demographics and driving experience.

Sex	Number	Age (yrs)	Height(m)	Weight(kg)	# Years Driving Experience	Driving Frequency (per week)	Daily Driving Duration (# Hrs) in Previous Year
Male	8	29.00 (8.35)	1.75 (0.10)	76.71 (14.26)	6.75 (11.44)	3.34 (3.67)	0.48 (0.28)
Female	8	26.50 (2.62)	1.72 (0.11)	68.04 (9.10)	3.44 (4.80)	11.83 (13.18)	0.95 (0.59)
All	16	27.75 (6.12)	1.73 (0.10)	72.38 (12.40)	5.09 (8.54)	7.59 (10.24)	0.72 (0.51)

### Conditions

**Fig 1:**
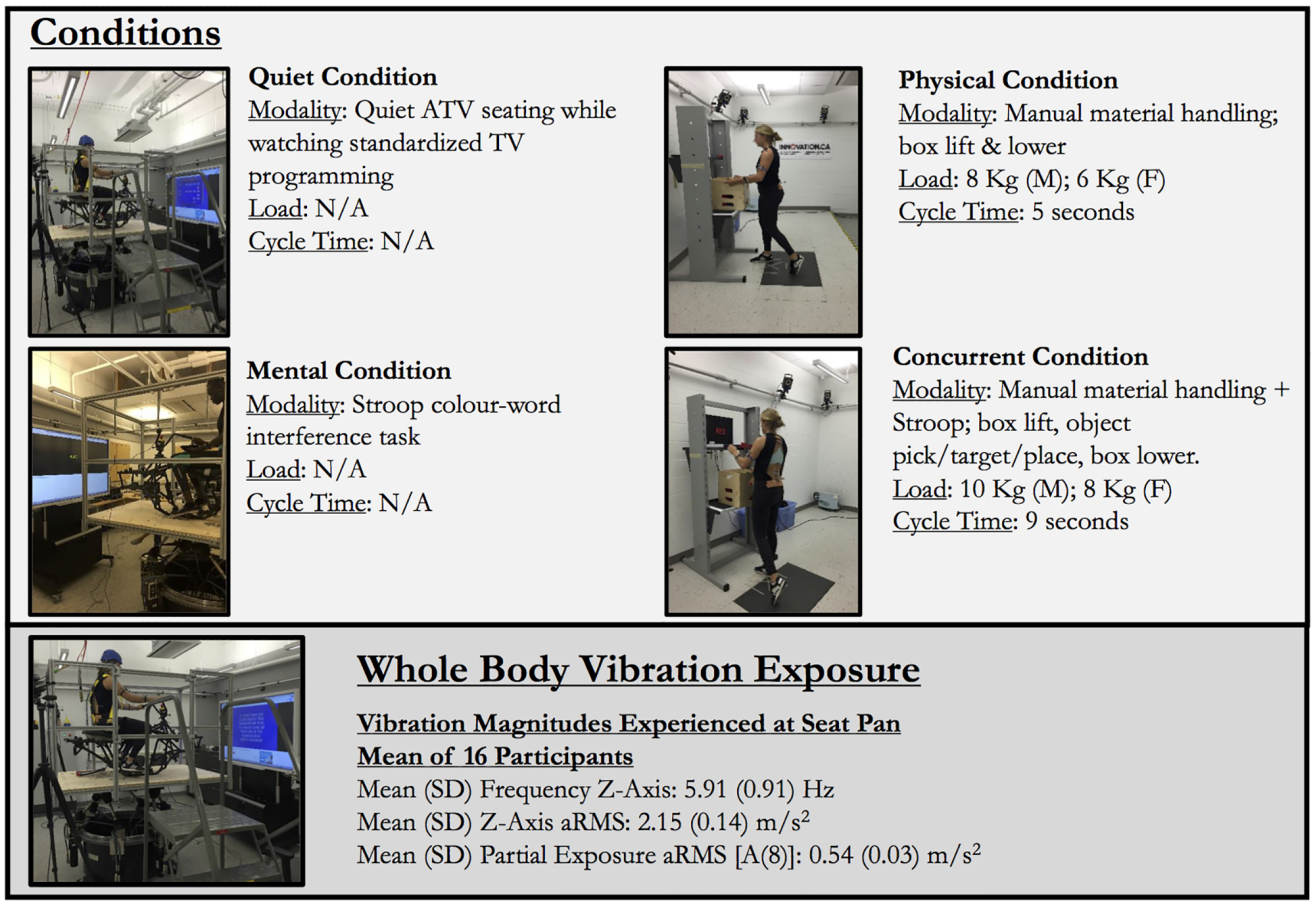
Experimental conditions and seated whole body vibration exposure.

Each participant performed four conditions on four separate days: (1) physically demanding work → WBV → physically demanding work, (2) mentally demanding work → WBV → mentally demanding work, (3) concurrent physical and mental work → WBV → concurrent physical and mental work, and (4) control quiet sitting → WBV → control quiet sitting (Fig 1). For the physical condition, participants were instructed to repetitively lift and lower a weighted box, from the floor to a shelving unit at the participant’s knuckle height. At a cycle time of 5 seconds, the maximum acceptable load was determined by psychophysical tables to accommodate 90% of the respective male and female working populations [24]. Using pre-determined task parameters (frequency: 1 lift every 5 seconds, box width: 34 cm, vertical distance: 76 cm, lift/lower between floor level and knuckle height), for multiple component manual material handling tasks, the maximum acceptable weight limit was calculated to be 8 kg and 6 kg for male and female participants, respectively. Participants were instructed to distribute their lift and lower actions, to the best of their ability, over the 5 second period. The mental condition required participants to perform a computerized Stroop colour-word interference task while seated on the stationary all-terrain vehicle (ATV). The Stroop test has been extensively used as a neuropsychological test to challenge cognitive-perceptual processes [25]. A third condition required participants to perform a manual material handling task and the Stroop test (concurrent condition). Based on psychophysical methods, the concurrent condition required lifting and lowering weighted boxes at a cycle time of 9 seconds. After lifting the weighted box to the shelving unit, at knuckle height, participants were asked to target, pick, and place coloured objects contained inside the box to colour-coded marked locations on a second shelf at shoulder level. Selection of the desired coloured object and designated location was determined based on the text colour of the Stroop test. The maximum acceptable weight was calculated using identical task parameters to the physically demanding task condition, with exception of the lifting frequency (1 lift every 9 seconds). Male and female participants were instructed to lift a total weight of 10 kg and 8 kg, respectively. Finally, participants were asked to sit on the stationary ATV (control condition) during the 30-minute period. In all sessions, during 30-minute WBV, participants watched standardized television programming to mitigate boredom effects. Practice time for each condition, at the discretion of the study facilitator, was provided for all participants prior to each session.

### Simulated whole body vibration

The occupational use of all-terrain vehicles has increased, particularly in the agricultural sector, for crop and livestock operations. Its increase use might coincide with changes in farming practices, where ATVs are preferred over traditional methods such as horses, tractors, and two-wheeled motorcycles [26]. In 2001, as many as 39% of all farms operating in the United States have reported ownership of one or more ATVs [27]. All-terrain vehicle operation is also pervasive in Canada’s agricultural sector. Lim and colleagues (2004) cited ATVs as the most common machines related to injury, superseding tractor usage [28]. One rationale is the increased usage of ATVs both for recreational and work activity.

Field-obtained z-axis accelerations, based on previously-collected ATV quad-bike riding data [29], were simulated using a six degree-of-freedom hexapod platform. Data available from the Dryad Digital Repository: https://doi.org/10.5061/dryad.9tr75. This data was obtained from rural workers in New Zealand, operating Yamaha King Quad 450 quad bikes. Rural workers completed a standardized route over typical farm terrain for a duration of approximately 30 minutes; the farm terrain consisted of mix surfaces (gravel, packed earth, soft earth, rough surfaces, ditches and stones) typical in grain and pulse cultivation, and livestock production. A four-minute sample of ATV acceleration datum that contained no mechanical shocks was extracted and used as the input signal into the rotopod. The signal was repeated continuously to form a 30-minute segment. To determine the actual resultant accelerations at the seat, a tri-axial accelerometer (16G, NexGen Ergonomics Inc., Quebec) was embedded into a rubber seatpad based on international standards (ISO 2631–1) and signals were processed and analyzed offline (Vibration Analysis ToolSet, NexGen Ergonomics Inc., Quebec). Measured data was processed using weighting filters (W_k_) described in ISO 2631–1 guidelines for seated vertical vibration. Vibration magnitude experienced by 16 participants are shown in Fig 1. The partial exposure A(8), which characterizes workday exposures of the frequency-weighted RMS acceleration by 8-hour extrapolation was found to be 0.54 m/s^2^ (0.03). Based on ISO 2631–1, over an 8-hour period, the observed partial A(8) was within the ISO health guidance caution zone (0.45–0.9 m/s^2^), indicating potential health risks.

### Fatigue test battery

A comprehensive set of complementary fatigue measures were selected based on previous studies that identified and evaluated measurements and detection methods that were reliable, responsive to various work demands, and linked to both health outcomes and system/operational performance [3, 30–31]. Additionally, to gain a comprehensive picture of fatigue development, measures reflecting changes within multiple systems and domains (biomechanical, physiological, cognitive, and sensory systems) were selected. Although fatigue involves a complex interaction between sites of impairment (e.g., central vs. peripheral fatigue), the primary focus of this study is to investigate general changes within different fatigue domains (e.g., physical, cognitive, visual). Understanding these general effects might help focus further investigations using measures indicating changes at central and peripheral locations. Therefore, the selected measures in this study may be indices of general changes that can be attributed to peripheral or central effects. For instance, maximum voluntary contraction force is often cited as the most direct assessment of physical fatigue, involving both central and peripheral processes [32]. Measurements were performed in quick succession and ordered to minimize possible residual effects between measures. All test batteries consisted of: Borg’s rating of perceived discomfort, postural sway, blink frequency, heart rate parameters (i.e., beats per minute—bpm), maximum voluntary contraction of the low back/lower extremity, and reaction time and accuracy based on choice reaction time (CRT) tests. Due to extensive time requirements to complete select tests, three measures were limited to pre- and post- session periods, and are indices of cumulative effects of both condition and WBV. These tests/tasks were: Semmes Weinstein monofilament test, Purdue pegboard assembly task, and psychomotor vigilance task (PVT). Data reduction, processing, and analysis were completed with conventional methods (Table 2) using MATLAB (Mathworks, Inc., Natick, MA, USA). Pre- and post-test batteries required approximately 20 minutes to complete while intermediate test batteries between first bout condition and seated WBV (TB01) and between seated WBV and second bout condition (TB02) tests required less than 10 minutes. Ample practice time (i.e., at least 10 trials) was provided prior to all tests, but additional time was granted at the discretion of the study facilitator, at the beginning of the entire experiment and at the beginning of each session.

**Table 2:** Summary of test battery collection strategy & processing/analysis methods.

Collection Strategy	Test Battery Measure	Collection Notes	Data Processing/Analysis	Interpretation Based on Increasing Workload or Fatigue
Every Interval (4 periods)	Borg’s Rating of Perceived Exertion (6–20)	Seven body parts: Lower back, hands/arms, neck, upper back, buttock, knee, ankle.	Expressed as value between 6 and 20. Submitted to non-parametric statistical tests	Increase in rating of perceived exertion (RPE) strongly coupled to fatigue [30].
	Postural Sway	Two-minute collection duration [33]. Arms at the sides, feet shoulder width apart, toes pointing forward. Participants instructed to stand as still as possible.	Root mean square displacement amplitude calculated in the anterior-posterior direction over middle 60-seconds [33].	Increase in sway (COP RMS displacement in A-P direction) associated with fatigue [34].
	Blink Frequency	Eye blink frequency (blinks/minute) during 2-minute collection, concurrent to postural sway. Participants instructed to gaze forward at a wall target.	High-pass filtered (0.1 Hz cut-off, 4^th^ Order Butterworth), to remove amplifier DC offset. Low-pass filtered (dual-pass, 4^th^ Order Butterworth, 10Hz cut-off), to removed EMG-based activity. Blinks determined by a threshold criterion described in [31].	Increase in eye blink rate & duration are indices of decrement in vigilance and reduced alertness [35].
	Heart Rate and Heart Rate Variability	Expressed as the number of beats per minute during 2-minute collection, concurrent to postural sway and eye blinks. Ratio of low frequency power band (0.04 to 0.15 Hz) and high frequency power band (0.15 to 0.40 Hz)–LF/HF.	Heart Rate = Frequency count. HRV = LF/HF ratio.	Decrease in HR indicative of lowered alertness. Increase in HR related to increase workload. Increase in HRV (LF/HF ratio) associated with mental tasks and fatigue [36]
	Maximum Voluntary Contraction (MVC)	Three 5-second MVC using fabricated back/lower limb force measurement system. Participants asked to sustain maximum exertion for 3-seconds with gradual increasing/decreasing ramps. Two-minute rest between contractions.	Resultant signal of the force along x, y, z axes. Resultant signal low-pass filtered (10 Hz, dual pass, 2^nd^ Order Butterworth). MVC force determined as the peak value of the three trials.	Cited as a direct assessment of neuromuscular fatigue. Decrease in MVC force correlated with increasing neuromuscular fatigue [32]
	Choice Reaction Time (CRT)	Ten consecutive trials. Four choices based on visual stimuli at random interval time between 1 and 4 seconds [37]. Input trial timeout of 2 seconds. Accuracy based on selection of correct choice and precision of targeting inner button. Multi-Operational Apparatus for Reaction Time (Lafayette Instrument).	Reaction time and number of errors (accuracy).	Increase reaction time and number of errors with increasing mental fatigue [38].
Pre- and Post- Session	Psychomotor Vigilance Task (PVT)	Standardized 10-minute trial. Visual stimuli presented at a variable interval of 2 to 10 seconds. Participants instructed to respond to the appearance of the LED stimulus with the thumb of their dominant hand.	Measurement parameters: %Errors (# errors committed/total number of trials), mean reaction time, reciprocal reaction time (response speed: 1/(RT/1000)), mean fastest 10% reaction time, and slowest 10% of reaction time.	PVT metrics related to lapses and psychomotor speed. Increases in errors and decrease in reaction time with increasing fatigue (time on task or sleep loss) [39]. Reciprocal transform sensitive to total and partial sleep loss.
	Purdue Pegboard Task	Two, 30-second trials for dominant and non-dominant hands, with instructions to insert pins into the pegboard, as quickly and accurately as possible. One, 60-second trial requiring assembly of pegs, two washers, and collars, using both hands as quickly and accurately as possible.	Number of inserted pins or completed assemblies.	Decrease in completed assemblies or inserted pins related to decrements in hand dexterity (fine motor) skills [40].
	Semmes Weinstein Monofilament Test	Sensory measurement taken on the sole of the dominant hand and foot. Locations marked with indelible felt tip pen at the beginning of each session. Limb supported and participant blindfolded. Starting with 2.83 monofilament, filament applied to 5 locations, varying location and time of application. Three touches considered as a single test, and participants verbally indicated the sensation of a perceived touch.	Smallest perceived monofilament diameter was recorded for each hand and foot location.	Correlation between altered plantar sensitivity and balance disorders. A reliable measure of cutaneous sensation [41].

### Experimental protocol

**Fig 2:**
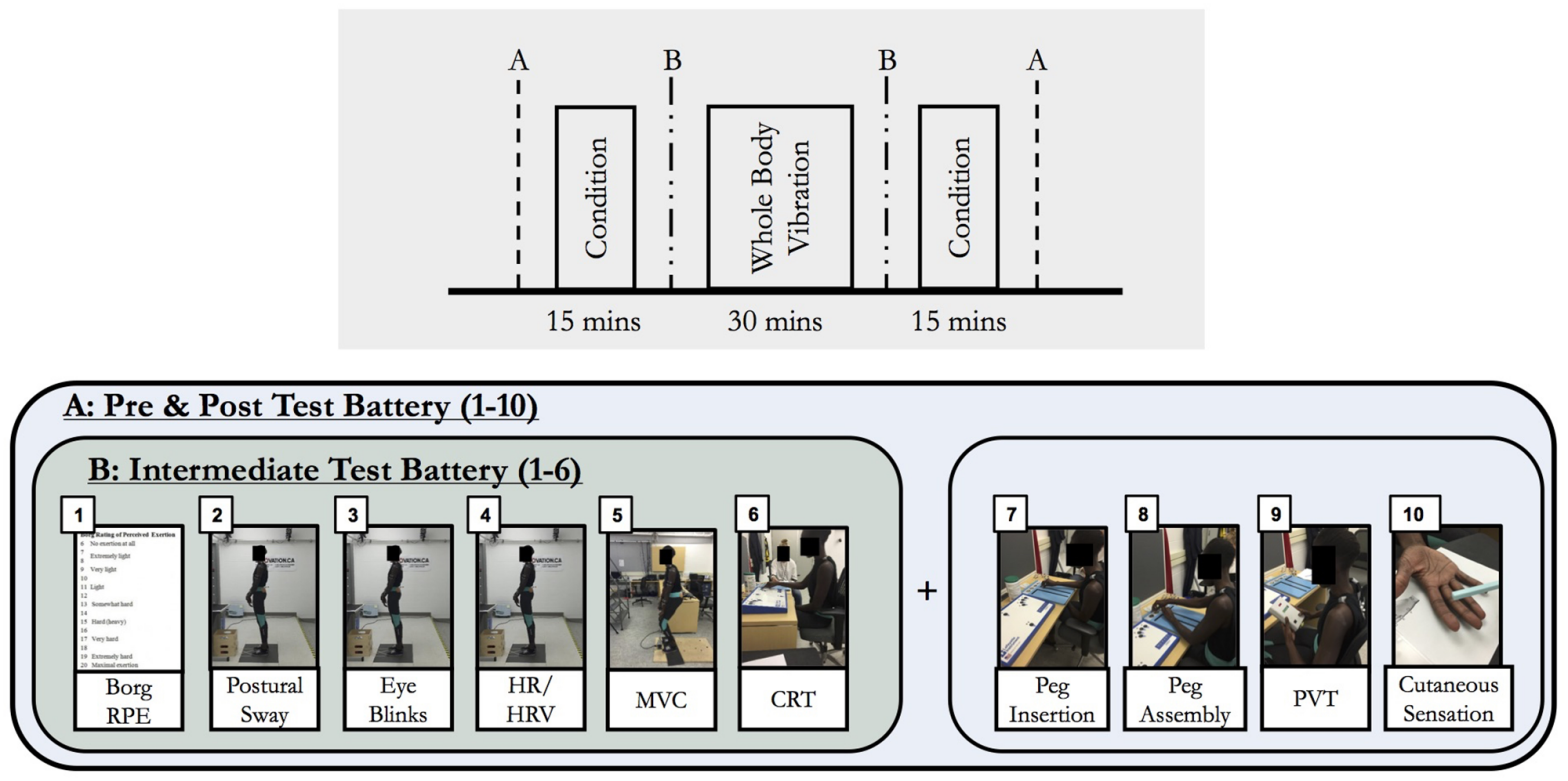
Experimental protocol. Top Inset: Experimental study collection protocol. Conditions presented in 2, 15-minute bouts, WBV in a single 30-minute bout. Bottom Inset: (A) Pre- and Post- session baseline test battery, (B) Intermediary test batteries between first bout of condition and ATV simulation and between ATV simulation and second bout of condition.

During each separate test session, an experimental condition was performed continuously in two-15 minute bouts, before and after seated WBV exposure (Fig 2). Separation of the 30-minute condition duration into two equal 15-minute bouts, before and after seated WBV exposure, helped identify possible effects of WBV on physical, mental, and concurrent tasks.

Previously-published findings had estimated total daily WBV exposure of 2.43 hours in agricultural herding work [42]. In a full-day field study, the average duration of continuous ATV driving from rural workers was 30 minutes [21]. This 30-minute WBV period has been previously selected to represent a typical continuous occupational operation of an ATV [43]. Similarly, in this study, participants were exposed to 30 minutes of ATV simulated accelerations.

The battery of fatigue measures was recorded at four periods: (a) beginning of the session (baseline), (b) after the first 15-minute task condition (TB01), (c) after 30-minutes of simulated WBV (TB02), and (d) after the second 15-minute task condition (Post). Collecting fatigue effects and responses with a test battery has advantages over collecting responses during activity on a simultaneous or continuous basis. Although continuous measurement might provide responses indicative of workload, a test battery allows for standardization and subsequently generalization across different work settings; it can also be administered without disruption to work processes. With this experimental protocol, we investigated the cumulative fatigue effects as a result of condition and/or seated WBV exposure.

### Statistical analyses

For each measure in each condition, measurement data at the four time periods were evaluated for their normality by plotting data as a histogram, drawing Q-Q plots, and by Shapiro-Wilk test. If data deviated from a Gaussian distribution, non-parametric approaches (i.e., Friedman’s test) were considered. Otherwise, data were submitted to a repeated measures analysis using a general linear mixed model approach to determine whether there were statistically significant time effects. In the event of a significant main effect, to determine significant paired comparisons, Tukey-Kramer post hoc tests followed repeated measures analysis (⍺ = 0.05, parametric), and Wilcoxon-signed-rank tests with Bonferroni correction (adjusted ⍺ = 0.0083, non-parametric) followed Friedman’s test. A two-way repeated measure mixed model analysis was performed on data from Purdue pegboard task (time x hand) and Semmes Weinstein monofilament tests of the sole of the dominant foot (time x foot test location). All statistical tests were performed using Statistical Analysis Software (Version 9.4, SAS Institute, Inc., Cary, NC, USA).

## Results

**Fig 3:**
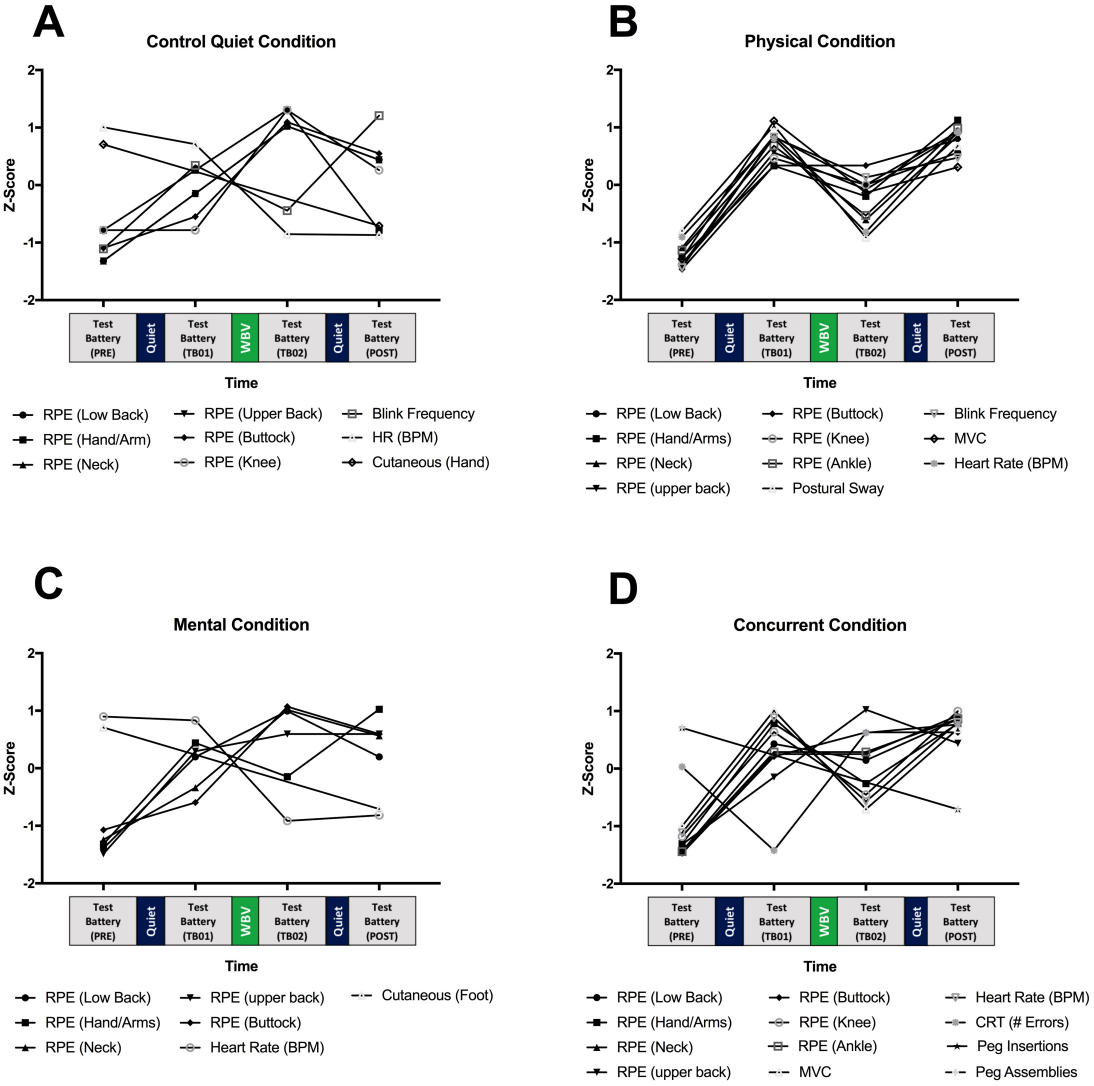
Visualization of trends of measures demonstrating statistically significant time effects. Values standardized to z-values to visualize trends of measures for each condition. Data plotted over time at intervals of time at which a test battery was collected (cutaneous sensation and Purdue pegboard test limited to Pre-and Post). Increase z-value score indicate increasing fatigue based on conventional interpretation of each measure. Decrease z-value score indicate decreasing fatigue. (A) Control Condition. (B) Physical Condition. (C) Mental Condition. (D) Concurrent Condition. Statistical post-hoc comparisons between time periods were shown in Table 3.

Statistically significant time effects (Table 3) were observed during control quiet (9 measures), physical (11 measures), mental (7 measures), and concurrent conditions (12 measures). Of these measures, 4 were unique (i.e., different measurement tools) in the control quiet condition. Five measures were unique in the physical condition, 3 in the mental condition, and 5 were unique measures in concurrent. To help visualize the trends of the statistically significant measures, the mean responses at each measurement period were standardized to a z-score and plotted in Fig 3. Of the statistically significant time effect measures, a frequent measure demonstrating significant changes was Borg’s rating of perceived exertion. Heart rate frequency also demonstrated significant trends in all conditions. Generally, trends of significant measures during the control condition indicate increasing self-perceived discomfort after the first 15-minute bout of quiet sitting and after 30-minutes seated WBV. Conversely, there were significant decreases in blink frequency and heart rate after seated WBV. Cutaneous sensation of the hand improved at the end of the session when compared to baseline values (Fig 3A). Self-perceived discomfort increased after both 15-minute bouts of physical activity, but decreased after subsequent WBV exposure. This trend was also observed with postural sway, blink frequency, and heart rate. Back/lower extremity MVC force significantly decreased as a result of physical activity, but increased after WBV (Fig 3B). The mental condition led to significant increases in body discomfort after the first 15-minute bout of the seated Stroop-test, and increased after WBV. Similar to the control condition, heart rate improved after seated WBV and cutaneous sensitivity improved at cessation compared to baseline (Fig 3C). Lastly, the concurrent condition trends were generally similar to the physical condition (Fig 3D); discomfort and heart rate increased after the two 15-minute concurrent bouts and decreased after WBV. MVC force decreased after the bouts of concurrent activity and increased after WBV. The Purdue pegboard test showed a decrease in the number of errors after seated WBV and the second bout of concurrent activity, when compared to the first bout of activity. Pre- and post- measures indicated an increase number of peg insertions and assemblies at the cessation of the session.

**Table 3:** Measures exhibiting significant time effects in four conditions. Mean(SD) or Median(interquartile range).

	Measure	Baseline	TB01	TB02	Post	Statistical Output
Control	Borg RPE (Lower Back)	6.00 (6–11)	8.00 (6–15)*	10.00 (6–15)*%	9.00 (6–14)*	χ2(3) = 29.132; p<0.0001
	Borg RPE (Hands/Arms)	6.00 (6–8)	**7.00 (6–11)**	8.00 (6–15)*%	6.00 (6–12)*	χ2(3) = 22.569; p<0.0001
	Borg RPE (Neck)	6.00 (6–8)	7.00 (6–11)*	8.00 (6–13)*%	7.50 (6–12)*	χ2(3) = 28.156; p<0.0001
	Borg RPE (Upper Back)	6.00 (6–12)	**7.00 (6–14)**	8.00 (6–14)*%	7.50 (6–15)*%	χ2(3) = 24.758; p<0.0001
	Borg RPE (Buttock)	6.00 (6–10)	**6.50 (6–12)**	8.00 (6–12)*	7.50 (6–12)*	χ2(3) = 22.1613; p<0.0001
	Borg RPE (Knee)	6.00 (6–8)	6.00 (6–11)	7.00 (6–12)*	6.50 (6–12)	χ2(3) = 13.4211; p = 0.0038
	Blink Frequency (per minute)	19.69 (10.43)	**29.2 (19.76)**	24.06 (18.06)	34.94(23.27)*%	F = 5.89; p = 0.0018; ηp2 = 0.2821
	Heart Rate (BPM)	89.43 (17.15)	88.30 (15.38)	82.33 (12.21)*%	82.27 (12.52)*%	F = 11.31; p<0.0001; ηp2 = 0.4469
	Semmes Weinstein (Hand All Locations)	2.94 (0.15)		2.89 (0.22)*		F = 4.34; p = 0.05; ηp2 = 0.0342
Physical	Borg RPE (Lower Back)	6.00 (6–11)	14.00 (6–20)*	11.00 (6–19)*%	13.00 (7–20)*#	χ2(3) = 37.033; p<0.0001
	Borg RPE (Hands/Arms)	6.00 (6–11)	9.00 (6–19)*	8.00 (6–19)*	10.50 (6–20)*#	χ2(3) = 32.919; p<0.0001
	Borg RPE (Neck)	6.00 (6–10)	10.00 (6–19)*	7.00 (6–19)*	10.00 (6–19)*	χ2(3) = 30.899; p<0.0001
	Borg RPE (Upper Back)	6.00 (6–11)	12.00 (7–20)*	10.00 (6–17)*%	13.00 (6–20)*#	χ2(3) = 35.565; p<0.0001
	Borg RPE (Buttock)	6.00 (6–10)	10.00 (6–19)*	10.00 (6–17)*	11.00 (6–18)*	χ2(3) = 35.565; p<0.0001
	Borg RPE (Knee)	6.00 (6–10)	10.00 (6–19)*	9.00 (6–18)*	11.00 (6–19)*	χ2(3) = 28.9112; p<0.0001
	Borg RPE (Ankle)	6.00 (6–12)	9.00 (6–19)*	7.00 (6–17)*	9.50 (6–17)*	χ2(3) = 22.6425; p<0.0001
	Postural Sway (mm)	1.51 (1.04)	3.27 (2.39)*	1.40 (0.80)%	2.93 (1.99)*#	F = 8.24; p = 0.0002; ηp2 = 0.3642
	Blink Frequency (per minute)	22.15 (15.72)	31.00 (22.91)*	28.27 (19.52)	**29.61 (22.48)**	F = 3.07; p = 0.0374; ηp2 = 0.1742
	MVC Force (N)	278.85 (138.57)	237.00 (123.21)*	258.81 (143.10)	**251.00 (133.30)**	F = 2.69; p = 0.05; ηp2 = 0.1641
	Heart Rate (BPM)	88.09 (16.36)	106.72 (20.65)*	89.03 (16.37)%	107.95 (20.03)*#	F = 28.09; p<0.0001; ηp2 = 0.6568
Mental	Borg RPE (Lower Back)	7.00 (6–13)	**10.00 (6–13)**	11.50 (6–15)*%	10.00 (6–15)*	χ2(3) = 21.414; p<0.0001
	Borg RPE (Hands/Arms)	6.00 (6–16)	7.50 (6–16)*	7.00 (6–14)	**8.00 (6–14)**	χ2(3) = 15.991; p = 0.0011
	Borg RPE (Neck)	6.00 (6–16)	7.00 (6–16)*	**8.50 (6–14)**	8.00 (6–15)	χ2(3) = 19.958; p = 0.0002
	Borg RPE (Upper Back)	6.00 (6–16)	9.00 (6–16)*	**9.50 (6–14)**	9.50 (6–15)	χ2(3) = 18.7099; p = 0.0003
	Borg RPE (Buttock)	6.00 (6–16)	7.00 (6–16)*	**10.50 (6–14)**	9.50 (6–15)	χ2(3) = 18.8857; p = 0.0003
	Heart Rate (BPM)	87.57 (15.00)	87.37 (11.19)*	82.03 (10.49)%	82.33 (7.61)*%	F = 6.19; p = 0.0014; ηp2 = 0.3065
	Semmes Weinstein (Foot All Locations)	3.31 (0.32)		2.91 (0.35)*		F = 4.70; p = 0.0467; ηp2 = 0.0654
Concurrent	Borg RPE (Lower Back)	6.50 (6–12)	13.00 (6–16)*	12.00 (6–17)*	14.50 (6–19)*	χ2(3) = 29.715; p<0.0001
	Borg RPE (Hands/Arms)	6.00 (6–12)	10.00 (6–14)*	8.00 (6–15)*	10.00 (6–18)*#	χ2(3) = 28.741; p<0.0001
	Borg RPE (Neck)	6.00 (6–13)	8.50 (6–14)*	8.50 (6–15)*	9.50 (6–17)*	χ2(3) = 26.885; p<0.0001
	Borg RPE (Upper Back)	6.00 (6–12)	**7.00 (6–14)**	8.00 (6–14)*	7.50 (6–15)*	χ2(3) = 24.758; p<0.0001
	Borg RPE (Buttock)	6.00 (6–12)	10.00 (6–14)*	11.00 (6–16)*	11.00 (6–17)*%	χ2(3) = 33.378; p<0.0001
	Borg RPE (Knee)	6.00 (6–14)	8.50 (6–15)*	7.00 (6–16)*	9.00 (6–19)*#	χ2(3) = 30.7674; p<0.0001
	Borg RPE (Ankle)	6.00 (6–11)	7.50 (6–14)*	7.50 (6–17)	8.00 (6–19)*	χ2(3) = 21.5481; p<0.0001
	MVC Force (N)	83.33 (11.69)	106.00 (16.04)*	89.50 (14.50)	**105.10 (18.46)**	F = 3.13; p = 0.0349; ηp2 = 0.1725
	Heart Rate (BPM)	690.90 (92.42)	578.10 (97.85)*	685.63 (107.44)%	587.67 (108.22)*#	F = 23.04; p<0.0001; ηp2 = 0.6220
	CRT (# or Errors)	1.57 (1.69)	0.86 (1.23)	**1.86 (1.29)**%	**1.93 (1.69)**%	F = 2.96; p = 0.04; η_p_^2^ = 0.1853
	Purdue Pegboard (R&L # Peg Insertions)	15.17 (1.72)		16.07 (1.76)*		F = 5.38; p = 0.036; ηp2 = 0.1774
	Purdue Pegboard (# Assemblies)	8.00 (1.73)		9.13 (1.68)*		F = 19.64; p = 0.0006; ηp2 = 0.5838

Dark box = increasing fatigue/workload response; intermediate shaded box = decreasing fatigue/workload response; white box = no substantial change from *preceding* test battery. Increase/decrease fatigue/workload response based on conventional interpretation (see Table 2).

* p<0.05 (0.0083) vs Baseline

^%^ p<0.05 (0.0083) vs TB01

^#^ p<0.05 (0.0083) vs TB02

## Discussion

This study led to six general findings: (1) Seated WBV exposure might provide a physical, sensorimotor, and physiological rest after physically demanding and concurrent physical/mental demands, but was not sufficient to recover to baseline values; (2) In all conditions seated WBV led to a decrease in heart rate; (3) Self-perceived discomfort significantly increased when compared to baseline values in almost all conditions and the majority of body locations, particularly after WBV exposure; (4) Improved cutaneous sensation of the dominant hand or foot in both control and mental conditions, although these changes were not observed during physical or concurrent sessions; (5) In the concurrent condition, manual dexterity speed (Purdue pegboard test) improved but the rate of errors during a CRT increased; in the control condition choice reaction time tests demonstrated moderate effect with faster reaction time but at the expense of increased accuracy errors; and (6) In both physical and control conditions, blink frequency decreased after seated WBV in comparison to 15-minute bouts of each condition.

Overall findings suggest that the effects of seated WBV, in combination with physical, mental, or concurrent tasks, are not additive but possibly synergistic or antagonistic. There appears to be a beneficial effect of seated WBV as a means of increasing task variation; but since excessive seated WBV may independently pose a health risk in the longer-term, these beneficial results may not be sensible as a long-term solution. The following sections discuss in greater detail the findings observed in each condition.

### Control quiet condition

Self-perceived exertion reflects a combination of fatigue and discomfort. Self-perceived exertion of the lower back and neck increased after the first bout of quiet sitting. There is extensive literature linking prolonged sitting and body discomfort (such as [44–45]). For instance, Waongenngarm and colleagues (2015) [46] found increases in discomfort at the neck, upper back, low back, and hip/thighs after an hour of upright, slumped, and forward leaning sitting postures.

After seated WBV exposure, discomfort of six body locations, including lower back and neck, were significantly higher than baseline. Discomfort of the lower back, neck, hands/arms, upper back) were significantly higher after seated WBV when compared to the first 15-minute bout of quiet sitting. At all body locations, the second bout of quiet sitting decreased discomfort, compared to seated WBV, but remained significantly higher than baseline values. WBV alone has also been shown to increase body discomfort, and when combined with a prolonged seating posture, the development of discomfort may be accelerated [47].

Blink frequency increased during bouts of quiet sitting. Increased blink frequency may be related to lower arousal levels or induced anger, excitement, and muscle tension [48]. Increases in blink rate may be a function of time-on-task, particularly repetitive and monotonous tasks that lead to boredom and fatigue [48]. Although this study provided standardized television programming (i.e., a quiz show) to reduce task monotony, it is possible that this strategy was not sufficient to mitigate boredom during bouts of quiet sitting. Sakamoto and colleagues (2010) [49] investigated the effects of viewing distance and visual content on physiological responses including blink frequency. When providing “everyday TV programs” and on-screen text to prevent sleepiness and boredom, post-test blink rates increased in all viewing distances when compared to baseline. Additionally, in this study, since English was not the first language for several participants, it is possible that the TV programme might have been insufficiently engaging.

We observed a slight decrease in blink frequency after 30-minutes of seated WBV compared to quiet sitting. The effects of WBV on alertness have been mixed. EEG studies found a reduction in wakefulness or alertness after WBV. In Satou et al., (2007) [50], the authors found that short-term exposure to periodic WBV (0.6 ms^-2^ r.m.s. at 10 Hz) led to a decrease in the Alpha Attenuation Coefficient (AAC), which is associated with low wakefulness. In another study, based on recordings of Beta and Theta brainwave spectrums, Azizan and colleagues (2014) [51] found decreases in beta wave activity during both low frequency random and sinusoidal WBV at 0.3m/s^2^ for 20 minutes. A decrease in Beta wave activity was more pronounced during the sinusoidal condition, which implies a greater drowsiness effect during sinusoidal compared to random WBV. However, the effect on alertness may be time-dependent during random vibration, as the authors observed a significant increase in Theta wave activity (state of drowsiness) after random vibration. Indeed, in an earlier study, Landström and Lundström (1985) [52] found greater reductions in wakefulness during sinusoidal than random vibration.

Although trends were not statistically significant, we observed increases in postural sway after seated WBV during the control condition. Whole body vibration is commonly linked to tonic vibration reflex (TVR), which involves activation of muscle spindles, mediation of the neural signals by 1a afferents, activation of alpha motoneurons, and increase in motor unit recruitment [53–54]. It has been speculated that TVR might lead to increase in force exertion, alterations in force control, increases in tendon stress, and when long-duration exposures to vibration are repetitive, might contribute to muscle fatigue [55, 56]. Studies have demonstrated increases in postural sway after WBV exposure [57]. For instance, Martin and colleagues (1980) observed alterations of postural control after 30 minutes of vertical accelerations at 18 Hz and ±0.5 g [58]. Mani and colleagues (2010) [57] speculate possible mechanisms between seated WBV and postural disturbances, including biodynamic responses of the head, neck, and trunk that might alter sensory structures and systems (e.g., vestibular and visual). However, Martin et al., (1980) [58] argued that postural changes may be better attributed to sensorimotor systems located in the legs rather than vestibular organs and vestibulospinal systems. In these studies, effects were observed when WBV was delivered at frequencies higher than the current study (5.91 Hz), which were based on seated quad bike WBV data. Earlier field evaluations of ATV quad bike WBV indicate that peak frequencies ranged between 4 and 6 Hz [59]. Interestingly, based on WBV exposure at comparable frequencies, Mani and colleagues (2015) [43] did not find significant postural control alterations immediately after ATV riding but significant increases on a second test battery following the first 10-minute test battery.

### Physical condition

Trends of perceived discomfort measures were consistent: an increase in fatigue response after the first bout of physical activity, a decrease after seated WBV exposure, followed by an increase in fatigue after the second 15-minute bout of physical activity. Although body discomfort decreased after seated WBV, when compared to the first bout of physical activity, discomfort of all body areas remained significantly higher than baseline. Therefore, seated WBV provided an opportunity to rest after a physically demanding task, but did not fully recover to baseline values.

Postural sway in the anterior-posterior direction increased after both bouts of physical activity when compared to baseline. After physical activity, increases in postural sway may be due to a correction of the central nervous system to account for increases in sensory threshold or reduced integration capacity [60]. Fatigue contributes to reduced sensory input and motor output of the postural system [34]. After seated WBV, postural sway significantly decreased. The decrease in postural sway may be better explained by the provisions of rest, which is further evidenced by maximum voluntary contraction force measurements. In this study, maximum strength of the back and lower extremities significantly decreased after the first bout of physical activity, slightly increased after seated WBV, and slightly decreased after the second bout. The increase in strength after seated WBV may be attributed to the provisions of rest since during the control condition, maximum strength did not increase after seated WBV.

Blink frequency and heart rate changed similarly to RPE, postural sway, and MVC force. Unlike the control condition, the increase in blink frequency during physical activity may be associated with increased muscle tension. The decrease in blink frequency after seated WBV could be attributed to the opportunity to reduce muscle tension. Heart rate was significantly elevated after physical activity and decreased to baseline values after WBV. Similar to the control condition, heart rate may be influenced by arousal levels. However, it is more likely that the observed significant reduction of heart rate was a consequence of reduced workload.

We can speculate from the above measurement responses that seated WBV introduces a form of task variation, providing an opportunity for physical rest following a physically demanding task. However, despite the opportunity to recover, exposure to WBV may prevent full recovery to baseline values.

### Mental condition

The mental condition revealed trends that closely resemble the control condition. Self-perceived discomfort increased after the Stroop test for five body locations, four of which experienced significantly higher discomfort than baseline. Lower back discomfort increased significantly after WBV relative to baseline and the first bout of mental work. Quite possibly, the increase in body discomfort during mental tasks may be due to reduced movement and prolonged static postures; this is associated with *bracing effort*, which may be prevalent in tasks requiring cognitive demand or psychological stress [61–62]. Berolo (2015) [61] demonstrated reductions in the duration and size of scapular movements and associated muscle activation with increasing cognitive demand. Although discomfort increased after seated WBV in comparison to the two bouts of mental work, this effect was not statistically significant.

Previous studies have demonstrated a heightened level of heart rate *during* the Stroop-colour word interference task (e.g., [63–64]). In this study, heart rate was collected *before* and *after* both 15-minute Stroop tasks and did not reveal elevated heart rate levels. Similar to the control condition, heart rate markedly decreased after seated WBV, indicative of reduced alertness, and remained depressed during the second bout of mental activity; but unlike the control condition, blink frequency did not markedly change. Therefore, since we did not observe a change in blink frequency, the Stroop task itself might have provided an engaging mental workload; however, over the *entire* test session, PVT parameters generally trended towards longer reaction times, indicating possible decrements in alertness by the end of the test session. Although we did not observe statistically significant differences, the mean slowest 10% reaction time parameter particularly led to a moderate effect, based on effect size calculation. Heart rate variability also demonstrated a moderate effect. HRV ratio, an indicator of sympathetic and parasympathetic balance, increased after the first bout of the Stroop test and further increased after seated WBV, but there was a slight decrease after the second bout, mirroring body discomfort. Mental tasks and fatigue is associated with a decrease in parasympathetic nerve activity (i.e., an enhancement of sympathetic nerve activity), and therefore an increase in LF/HF ratio [65].

During control and mental conditions, cutaneous sensitivity of the hands and feet, respectively, increased at the end of the entire test session compared to baseline. These results are not consistent with previous studies on cutaneous sensation after WBV exposure. Since much of this literature on cutaneous sensation and WBV focuses on vibration (30–45 Hz, transmitted through the feet) delivered for therapeutic or as an exercise modality (e.g., [66–67]), our findings suggest a need for further research to focus on occupationally relevant WBV exposures.

### Concurrent condition

The concurrent mental and physical condition resulted in significant time effects based on the following measures: discomfort, MVC force, heart rate, number of errors during choice reaction time test, and number of insertions and assemblies during the Purdue pegboard test. Self-perceived discomfort, MVC force, and heart rate parameter responses were similar to the physical condition. Unlike the physical condition, postural sway did not appear to significantly change over the four periods, however, there was a moderate effect of time. Trends indicated an increase in posture sway after the first bout of concurrent activity, a slight decrease after seated WBV, and an increase after the second bout to values approximating the first bout.

Apart from the upper back, discomfort of all body areas significantly increased after the first bout of concurrent activity. Discomfort of the upper back and buttock significantly increased after seated WBV when compared to the first 15-minutes of activity. Discomfort of all remaining body areas did not change or they decreased after seated WBV, but levels remained significantly higher than baseline. As with the physical condition, seated WBV might have provided a rest period to minimize bodily discomfort, but discomfort levels did not recover to baseline values possibly due to the discomfort-enhancing effects of seated WBV.

Maximum voluntary strength of the low back and lower extremities demonstrated a significant decrease after the first bout, an increase in strength approximating baseline values after WBV, and a decrease after the second bout. The increase in strength after WBV, like the physical condition, may be attributed to the opportunity to rest and possibly, albeit less likely, an increase in muscle tension as a result of tonic vibration reflex.

Heart rate responses significantly increased after the first 15-minutes of concurrent activity, a significant decrease after WBV, and a significant increase after the second 15-minutes of concurrent activity. It is probable, like the physical condition, that changes in heart rate was a consequence of changes in physical workload.

In contrast to the physical condition, the concurrent activity resulted in an increased number of accuracy errors committed during a choice reaction test, particularly after WBV. The number of pegboard insertions and assemblies, on the other hand, improved over the course of the test session. This might suggest that participants, after concurrent and WBV activities, preserved their reaction time (i.e., movement speed) but at the expense of committing increased number of errors. To support this, although CRT reaction time was not statistically significant, trends indicated lower reaction times towards the end of the session. Previous studies have documented changes to reaction time and error rates after WBV, and as described earlier, the control condition resulted in moderate effects of increased reaction time but at the expense of decreased accuracy. Tian and colleagues (1996) [68] also observed significant increases in visual motor reaction time but increases in error rates among female crane operators, pre- and post- work. Newell and Mansfield (2008) [69] found that WBV exposure had a significant effect on the number of CRT errors and asserted its effect on arousal and motivation, rather than changes to motor control, coordination, and information processing speed. In Newell and Mansfield (2008) [69], the authors attributed an increased level of WBV-induced frustration and resulting distraction as the primary cause to poor performance. Ljungberg and Neely (2007) [70] observed similar effects. In that study, participants performed the attention task significantly faster but committed more errors after three axes vibration at 1.1 m/s^2^ r.m.s., but participants reported higher ratings of self-perceived alertness. Whether WBV influences alertness or motivation, as Ljungberg and Neely (2007) [70] noted, “the combination of perceived alertness [*or changes in motivation*] while at the same time exhibiting degraded performance could be a dangerous combination.” (pp. 115, Ljungberg & Neely, 2007) [70].

### Implications for occupational applications

Like many industries with non-routine multifunctional work, it is unclear how agricultural workers should best arrange work tasks and schedule their activities. The present study demonstrates the complexity of risk assessment for non-routine work. WBV combined with different demands do not have simply additive effects, but are often synergistic or antagonistic. Arranging and scheduling work duties appears to be contingent on the inter-dependent demands of the tasks. The present findings supported the beneficial effect of task variation, even if the task consisted of WBV exposure or physical/mental demands that independently pose MSD risk. When tasks are physically demanding, seated WBV led to a decrease in self-perceived discomfort, reduced postural sway, and limited strength recovery. Tasks composed of concurrent physical and mental demands resulted in beneficial physical effects from a WBV interlude, but possible impairments to visual motor skills (i.e., accuracy errors). Whole body vibration alone might lead to increased self-perceived discomfort effects and might affect arousal or alertness. However, optimal task ratios remain unclear; although in the short-term there could be beneficial effects as a result of increased task variation, there may be an increased risk of MSD, including low back pain and injury, in the longer-term if demands are excessive.

## Strengths and limitations

One limitation of this study is the representativeness of the simulated WBV exposure, which was limited to a single axis stochastic signal excluding mechanical shocks. This was mainly due to technical limitations to reproduce motion with high precision. The representativeness of the WBV exposure might influence the observed sensorimotor responses. For example, previous field studies have demonstrated a significant effect of WBV on postural balance [57]; although the present study observed increases in postural sway, it did not meet the statistical criterion. It is quite possible that factors not replicated in this study might result in stronger trends that have been observed in naturalistic studies. These factors include mechanical shocks, increased exposure duration, multi-axis vibration and motion, and visual information in realistic ATV operation (i.e., exogenous factors). Nevertheless, the simulated WBV exposure approximated accelerations experienced in ATV operation at continuous driving durations typical in agricultural work [8, 21]. Future investigations should consider these factors and their contributions to fatigue effects. A second limitation is the ecological validity of the task demands simulated in this experiment. In this study, the frequency of manual material handling lifting was determined using established psychophysical guidelines, which considers productivity (i.e., as if performed on an incentive basis) and discomfort and fatigue (i.e., without experiencing unusual and undue discomfort if performed for an 8-hour period). Although it may be advantageous to invest in a detailed task analysis for occupational duties to calculate typical duty cycle, cycle time, and force exertions, the non-cyclical nature of these types of work may pose challenges in determining work parameters. A third limitation is the lack of a “no WBV” condition to help determine whether observed effects from seated WBV were a result of prolonged sitting or exposure to WBV. Based on overall seat comfort (or body discomfort), qualitative models describe both static seat factors (e.g., seat stiffness) and dynamic seat factors (e.g., WBV) as contributing characteristics [71]. Santos et al., (2008) investigated the effects of WBV on postural balance, reflex response, and muscle activity, and compared these effects against prolonged sitting without vibration [72]. Although muscle activities of the lower back were 22% to 48% higher after WBV compared to sitting alone, there were no extraordinary effects of WBV on balance and muscle reflex response [72]. From an injury aetiology perspective and to effectively develop targeted interventions, it might be important to disentangle the effects of prolonged sitting and WBV. However, the objective of *this* study was to understand the combined effects of relevant occupational exposures. In realistic work, exposure WBV is often delivered while seated during operation of vehicles and machinery. Finally, the addition of a test battery might unintentionally introduce a passive or active work break. There is a need to improve occupational monitoring of fatigue using a test battery; further research is required to help inform decisions on the selection, arrangement, and interpretation of fatigue measures.

A strength of this study is the inclusion of a complementary set of test battery measures to determine multidimensional changes associated with different work demands. Relying on observed changes to single dimensions or systems might not provide a comprehensive picture of fatigue development. Finally, contemporary jobs have been increasingly less monotask and more difficult to define by conventional work parameters (i.e., cycle time, duty cycle). This study provides an initial contribution to a sparse literature in non-routine work consisting of multiple ergonomic demands; however further research should be devoted to understanding the complex interactions between these demands.

## Conclusion

Jobs in agricultural, construction, transportation, and forestry are characterized as non-routine, non-cyclical, peripatetic, with discretionary and non-discretionary work breaks, involving physically and mentally demanding work tasks and frequent exposure to WBV. When seated WBV exposure was introduced to physical or concurrent demanding tasks, there was an observed beneficial effect in terms of self-perceived discomfort, strength, and postural sway. In the concurrent task, although there were beneficial physical effects after seated WBV, there were possible impairments to visual motor skills. Seated WBV without sequential demands may lead to increased bodily discomfort and affect the operator’s arousal and alertness. Therefore, as a work arrangement strategy, there is utility in seated vehicle operation as a task variation strategy, but is dependent on the combination of demands and may independently pose long-term health risks.

## References

[1] Mathiassen SE, Christmansson M. Variation and autonomy In: Delleman NJ, Haslegrave CM, Chaffin DB, editors. Working postures and movements: Tools for evaluation and engineering. Boca Raton: CRC Press; 2004 p. 330–355.

[2] Gold JE, Park J-S, Punnett L. Work routinization and implications for ergonomic exposure assessment. Ergonomics. 2006; 49(1): 12–27. doi: 10.1080/00140130500356643 16393801 10.1080/00140130500356643

[3] Yung M, Bigelow PL, Hastings DM, Wells RP. Detecting within- and between-day manifestations of neuromuscular fatigue at work: An exploratory study. Ergonomics, 2014; 57(10): 1562–1573. doi: 10.1080/00140139.2014.934299 24998392 10.1080/00140139.2014.934299

[4] Jahncke H, Hygge S, Mathiassen SE, Hallman D, Mixter S, Lyskov E. Variation at work: Alterations between physically and mentally demanding tasks in blue-collar occupations. Ergonomics, 2017. 10.1080/00140139.2017.1282630 28112588

[5] Davis KG, Kotowski MS. Understanding the ergonomic risk for musculoskeletal disorders in the United States agricultural sector. American Journal of Industrial Medicine. 2007; 50(7): 501–511. doi: 10.1002/ajim.20479 17506508 10.1002/ajim.20479

[6] McMillan M, Trask C, Dosman J, Hagel L, Pickett W. Prevalence of musculoskeletal disorders among Saskatchewan farmers. Journal of Agromedicine. 2015; 20(3): 292–301. doi: 10.1080/1059924X.2015.1042611 26237719 10.1080/1059924X.2015.1042611

[7] Waldenström M, Josephson M, Persson C, Theorell T. Interview reliability for assessing mental work demands. Journal of Occupational Health Psychology. 1998; 3(3): 209–216. 9684212 10.1037//1076-8998.3.3.209

[8] Milosavljevic S, Bergman F, Rehn B, Carman AB. All-terrain vehicle use in agriculture: Exposure to whole body vibration and mechanical shock. Applied Ergonomics. 2010; 41: 530–535. doi: 10.1016/j.apergo.2009.11.002 19944407 10.1016/j.apergo.2009.11.002

[9] Armstrong TJ, Buckle P, Fine LJ, Hagberg M, Jonsson B, Kilbom A, et al A conceptual model for work-related neck and upper-limb musculoskeletal disorders. Scandinavian Journal of Work, Environment, and Health. 1993; 19: 73–84 10.5271/sjweh.1494 8316782

[10] Nahrgang JD, Morgeson FP, Hofmann DA. Safety at work: A meta-analytic investigation of the link between job demands, job resources, burnout, engagement, and safety outcomes. Journal of Applied Psychology. 2011; 96(1): 71–94. doi: 10.1037/a0021484 21171732 10.1037/a0021484

[11] Boksem MA, Meijman TF, Lorist MM. Effects of mental fatigue on attention: an ERP study. Cognitive Brain Research. 2005; 25(1): 107–116. doi: 10.1016/j.cogbrainres.2005.04.011 15913965 10.1016/j.cogbrainres.2005.04.011

[12] Mehta RK, Agnew MJ. Influence of mental workload on muscle endurance and recovery during intermittent static work. European Journal of Applied Physiology. 2012; 112: 2891–2902. doi: 10.1007/s00421-011-2264-x 22143842 10.1007/s00421-011-2264-x

[13] Davis KG, Marras WS, Heaney CA, Waters TR, Gupta P. The impact of mental processing and pacing on spinal loading– 2002 Volvo Award in Biomechanics. Spine. 2002; 27(23): 2645–2653. 12461390 10.1097/00007632-200212010-00003

[14] Yung M, Manji R, Wells RP. Exploring the relationship of task performance and physical and cognitive fatigue during a daylong light precision task. Human Factors. 2017. Forthcoming. 10.1177/0018720817717026 28658591

[15] Lings S, Leboeuf-Yde C. Whole-body vibration and low back pain: a systematic, critical review of the epidemiological literature 1992–1999. International Archives of Occupational and Environmental Health. 2000; 73(5): 290–297. 10963411 10.1007/s004200000118

[16] Wikström B-O, Kjellberg A, Landström U. Health effects of long-term occupational exposure to whole-body vibration: A review. International Journal of Industrial Ergonomics, 1994; 14: 273–292.

[17] Young E, Kreiger N, Purdham J, Sass-Kortsak A. Prostate cancer and driving occupations: Could whole body vibration play a role? International Archives of Occupational and Environmental Health, 2009; 82(5): 551–556. doi: 10.1007/s00420-009-0403-z 19242718 10.1007/s00420-009-0403-z

[18] Pollard J, Porter W, Mayton A, Xu X, Weston E. The effect of vibration exposure during haul truck operation on grip strength, touch sensation, and balance. International Journal of Industrial Ergonomics, 2017; 57: 23–31. doi: 10.1016/j.ergon.2016.11.009 28220051 10.1016/j.ergon.2016.11.009 PMC5315416

[19] Ahuja S, Davis J, Wade LR. Postural stability of commercial truck drivers: Impact of extended duration. Proceedings of the 49th Annual Meeting of the Human Factors and Ergonomics Society; Orlando, FL; 2005.

[20] Lindberg E, Carter N, Gislason T, Janson C. Role of snoring and daytime sleepiness in occupational accidents. American Journal of Respiratory and Critical Care Medicine. 2001; 164(11): 2031–2035. doi: 10.1164/ajrccm.164.11.2102028 11739131 10.1164/ajrccm.164.11.2102028

[21] Milosavljevic S, McBride DI, Bagheri N, Vasiljev RM, Carman AB, Rehn B, et al Factors associated with quad bike loss of control events in agriculture. International Journal of Industrial Ergonomics. 2011; 41: 317–321.

[22] Thiffault P. Addressing human factors in the motor carrier industry in Canada. Canadian Council of Motor Transport Administrators (CCMTA). 2001.

[23] Du BB, Bigelow PL, Wells RP, Davies HW, Hall P, Johnson PW. The impact of different seats and whole body vibration exposures on truck driver vigilance and discomfort. Ergonomics. 2017. Forthcoming. 10.1080/00140139.2017.1372638 28845747

[24] Snook SH, Ciriello VM. The design of manual material handling tasks: Revised tables of maximum acceptable weights and forces. Ergonomics. 1991; 34(9): 1197–1213. doi: 10.1080/00140139108964855 1743178 10.1080/00140139108964855

[25] MacLeod CM. Half a century of research on the Stroop effect: An integrative review. Psychological Bulletin. 1991; 109(2): 163–203. 2034749 10.1037/0033-2909.109.2.163

[26] O’Connor T, Hanks H, Steinhardt D. All-terrain vehicle crashes and associated injuries in north Queensland: Findings from the rural and remote road safety study. The Australian Journal of Rural Health. 2009; 17(5): 251–256. doi: 10.1111/j.1440-1584.2009.01086.x 19785677 10.1111/j.1440-1584.2009.01086.x

[27] Goldcamp EM, Myers J, Hendricks K, Layne L, Helmkamp J. Nonfatal all-terrain vehicle-related injuries to youths living on farms in the United States. Children & Youth. 2001; 22(4): 308–313. 10.1111/j.1748-0361.2006.00051.x 17010027

[28] Lim GW, Belton KL, Pickett W, Schopflocher DP, Voaklander DC. Fatal and non-fatal machine-related injuries suffered by children in Alberta, Canada, 1990–1997. American Journal of Industrial Medicine. 2004; 45(2): 177–185. doi: 10.1002/ajim.10325 14748048 10.1002/ajim.10325

[29] Kociolek AM, Lang A, Trask C, Vasiljev RM, Milosavljevic S. Exploring head and neck vibration exposure from quad bike use in agriculture. International Journal of Industrial Ergonomics. 2017. Forthcoming.

[30] Yung M, Wells RP. Sensitivity, reliability and the effects of diurnal variation on a test battery of field usable upper limb fatigue measures. Ergonomics. 2017; 60(7): 923–939. doi: 10.1080/00140139.2016.1243734 27691721 10.1080/00140139.2016.1243734

[31] Yung M, Wells RP. Responsive upper limb and cognitive fatigue measures during light precision work: An 8-hour simulated micro-pipetting study. Ergonomics. 2017; 60(7): 940–956. doi: 10.1080/00140139.2016.1242782 27684480 10.1080/00140139.2016.1242782

[32] Vøllestad NK. Measurement of human muscle fatigue. Journal of Neuroscience Methods. 1997; 74(2): 219–227. 9219890 10.1016/s0165-0270(97)02251-6

[33] Carpenter MG, Frank JS, Winter DA, Peysar GW. Sampling duration effects on centre of pressure summary measures. Gait and Posture. 2001; 13: 35–40. 11166552 10.1016/s0966-6362(00)00093-x

[34] Paillard T. Effects of general and local fatigue on postural control: A review. Neuroscience and Biobehavioral Reviews. 2012; 36(1): 162–176. doi: 10.1016/j.neubiorev.2011.05.009 21645543 10.1016/j.neubiorev.2011.05.009

[35] Morris TL, Miller JC. Electrooculographic and performance indices of fatigue during simulated flight. Biological Psychology. 1996; 42: 343–360. 8652752 10.1016/0301-0511(95)05166-x

[36] Mizuno K, Kanako T, Watanabe Y, Kuratsune H. Fatigue correlates with the decrease in parasympathetic sinus modulation induced by a cognitive challenge. Behavioral and Brain Functions. 2014; 10(25). 10.1186/1744-9081-10-25 PMC4123830 25069864

[37] Jensen AR, Munro E. Reaction time, movement time, and intelligence. Intelligence. 1979; 3(2): 121–126.

[38] Rosa RR, Colligan MJ. Long workdays versus restdays: Assessing fatigue and alertness with a portable performance battery. Human Factors. 1988; 30(3): 305–317. doi: 10.1177/001872088803000305 3169791 10.1177/001872088803000305

[39] Lamond N, Dawson D, Roach GD. Fatigue assessment in the field: Validation of a hand-held electronic psychomotor vigilance task. Aviation, Space, and Environmental Medicine. 2005; 76(5): 486–489. 15892548

[40] Tiffin J, Asher EJ. The Purdue pegboard: Norms and studies of reliability and validity. Journal of Applied Psychology. 1948; 32(3): 234–247. 18867059 10.1037/h0061266

[41] Bell-Krotoski JA. Threshold detection and Semmes-Weinstein monofilaments. Journal of Hand Therapy. 1995; 8(2): 155–162. 7550627 10.1016/s0894-1130(12)80314-0

[42] Clay L, Milosavljevic S, Trask C. Predicting whole body vibration exposure from occupational quad bike use in farmers. Safety. 2015; 1(1): 71–83.

[43] Mani R, Milosavljevic S, Ribeiro DC, Sullivan SJ. Effects of agricultural quad bike driving on postural control during static, dynamic and functional tests–A field study. International Journal of Industrial Ergonomics. 2015; 50: 158–169.

[44] Vergara M, Page A. Relationship between comfort and back posture and mobility in sitting-posture. Applied Ergonomics. 2002; 33(1): 1–8 11827133 10.1016/s0003-6870(01)00056-4

[45] Søndergaard KHE, Olesen CG, Søndergaard EK, de Zee M, Madeleine P. The variability and complexity of sitting postural control are associated with discomfort. Journal of Biomechanics. 2010; 43(10): 1997–2001. doi: 10.1016/j.jbiomech.2010.03.009 20399433 10.1016/j.jbiomech.2010.03.009

[46] Waongenngarm P, Rajaratnam BS, Janwantanakul P. Perceived body discomfort and trunk muscle activity in three prolonged sitting postures. Journal of Physical Therapy Science, 2015; 27(7): 2183–2187. doi: 10.1589/jpts.27.2183 26311951 10.1589/jpts.27.2183 PMC4540846

[47] Mansfield N, Mackrill J, Rimell AM, MacMull SJ. Combined effects of long-term sitting and whole body vibration on discomfort onset for vehicle occupants. 2014; ISRN Automotive Engineering.

[48] Stern JA, Walrath LC, Goldstein R. The endogenous eyeblink. Psychophysiology. 1984; 21(1): 22–33. 6701241 10.1111/j.1469-8986.1984.tb02312.x

[49] Sakamoto K, Aoyama S, Asahara S, Yamashita K, Okada A. Evaluation of the effect of viewing distance on visual fatigue in a home viewing environment. Journal of Human Ergology. 2010; 39(1): 1–13. 21922786

[50] Satou Y, Ando H, Nakiri M, Nagatomi K, Yamaguchi Y, Hoshiko M, et al Effects of short-term exposure to whole-body vibration on wakefulness level. Industrial Health. 2007; 45: 217–223. 17485865 10.2486/indhealth.45.217

[51] Azizan MA, Fard M, Azari MF. Characterization of the effects of vibration on seated driver alertness. Nonlinear Engineering. 2014; 3(3): 163–168.

[52] Landström U, Lundström R. Changes in wakefulness during exposure to whole body vibration. Electroencephalography and Clinical Neurophysiology, 1985; 61(5): 411–415. 2412793 10.1016/0013-4694(85)91032-6

[53] Hagbarth KE. The effect of muscle vibration in normal man and in patients with motor disease In: Desmedt JE., editors. New developments in electromyography and clinical neurophysiology. Basel, Switzerland: Karger; 1973 p. 428–443.

[54] De Gail P, Lance JW, Neilson PD. Differential effects on tonic and phasic reflex mechanisms produced by vibration of muscles in man. Journal of Neurology & Neurosurgery and Psychiatry. 1966; 29(1): 1–11. 10.1136/jnnp.29.1.1 PMC495977 5910574

[55] Radwin RG, Armstrong TJ, Chaffin DB. Power hand tool vibration effects on grip exertion. Ergonomics. 1987; 30(5): 833–855.

[56] Park H-S, Martin BJ. Contribution of the tonic vibration reflex to muscle stress and muscle fatigue. Scandinavian Journal of Work, Environment & Health. 1993; 19: 35–42. 10.5271/sjweh.1506 8465170

[57] Mani R, Milosavljevic S, Sullivan SJ. The effect of occupational whole-body vibration on standing balance: A systematic review. International Journal of Industrial Ergonomics, 2010; 40: 698–709.

[58] Martin B, Gauthier GM, Roll JP, Hugon M, Harlay F. Effects of whole-body vibrations on standing posture in man. Aviation, Space, and Environmental Medicine. 1980; 51(8): 778–787. 7417144

[59] Milosavljevic S, McBride DI, Bagheri N, Vasiljev RM, Mani R, Carman AB, et al Exposure to whole-body vibration and mechanical shock: A field study of quad bike use in agriculture. The Annals of Occupational Hygiene. 2011; 55(3): 286–295. doi: 10.1093/annhyg/meq087 21220741 10.1093/annhyg/meq087

[60] Carpenter MG, Murnaghan CD, Inglis JT. Shifting the balance: evidence of an exploratory role for postural sway. Cognitive, Behavioral, and Systems Neuroscience. 2010; 171(1): 196–204. 10.1016/j.neuroscience.2010.08.030 20800663

[61] Berolo S. Demonstrating relationships between workplace demands and exposures related to musculoskeletal disorders and stress-related health outcomes [dissertation]. Waterloo (ON): University of Waterloo; 2015.

[62] Whatmore GB, Kohli DR. The physiopathology and treatment of functional disorders. New York, NY: Grune & Stratton, Inc 1974.

[63] Renaud P, Blondin J-P. The stress of Stroop performance: physiological and emotional responses to color–word interference, task pacing, and pacing speed. International Journal of Psychophysiology. 1999; 27(2): 87–97. 10.1016/s0167-8760(97)00049-4 9342640

[64] Boutcher YN, Boutcher SH. Cardiovascular response to Stroop: Effect of verbal response and task difficulty. Biological Psychology. 2006; 73(3): 235–241. doi: 10.1016/j.biopsycho.2006.04.005 16730405 10.1016/j.biopsycho.2006.04.005

[65] Mizuno K, Tanaka M, Yamaguti K, Kajimoto O, Kuratsune H, Watanabe Y. Mental fatigue caused by prolonged cognitive load associated with sympathetic hyperactivity. Behavioral and Brain Functions. 2011; 7(17). 10.1186/1744-9081-7-17 PMC3113724 21605411

[66] Sonza A, Robinson CC, Achaval M, Zaro MA. Whole body vibration at different exposure frequencies: Infrared thermography and physiological effects. The Scientific World Journal. 2015. 10.1155/2015/452657 PMC4310482 25664338

[67] Pollock RD, Provan S, Martin FC, Newham DJ. The effects of whole body vibration on balance, joint position sense and cutaneous sensation. European Journal of Applied Physiology, 2011; 111(12): 3069–3077. doi: 10.1007/s00421-011-1943-y 21455611 10.1007/s00421-011-1943-y

[68] Tian LG, Gang WL, Ping HJ, Ji LC, Fang LY. Investigation of eyestrain and working capacity for female crane operators. International Journal of Industrial Ergonomics. 1996; 18(2–3): 221–224.

[69] Newell GS, Mansfield NJ. Evaluation of reaction time performance and subjective workload during whole-body vibration exposure while seated in upright and twisted postures with and without armrests. International Journal of Industrial Ergonomics. 2008; 38: 499–508.

[70] Ljungberg JK, Neely G. Cognitive after-effects of vibration and noise exposure and the role of subjective noise sensitivity. Journal of Occupational Health. 2007; 49: 111–116. 17429168 10.1539/joh.49.111

[71] Ebe K, Griffin MJ. Qualitative models of seat discomfort including static and dynamic factors. Ergonomics. 2000; 43(6): 771–790. doi: 10.1080/001401300404742 10902887 10.1080/001401300404742

[72] Santos BR, Larivière C, Delisle A, Plamondon A, Boileau P-É, Imbeau D, et al A laboratory study to quantify the biomechanical responses to whole-body vibration: The influence on balance, reflex response, muscular activity and fatigue. International Journal of Industrial Ergonomics. 2008; 38: 626–639.

